# A Positive-Pressure Environment Disposable Shield (PEDS) for COVID-19 Health Care Worker Protection

**DOI:** 10.1017/S1049023X20000643

**Published:** 2020-05-13

**Authors:** Li-Chien Chien, Christian K. Beÿ, Kristi L. Koenig

**Affiliations:** 1. Disaster Division, Emergency Department, Taipei City Hospital; Institute of Hospital and Health Care Administration, National Yang-Ming University, Taipei City, Taiwan; 2. University of California San Diego, La Jolla, California USA; 3. County of San Diego, Health & Human Services Agency, Emergency Medical Services, San Diego, California USA; 4. University of California Irvine, Department of Emergency Medicine, Orange, California USA

**Keywords:** coronavirus, COVID-19, heath care worker protection, intubation, PPE

## Abstract

The COVID-19 pandemic has strained health care system resources and reduced the availability of life-sustaining and medical-grade personal protective equipment (PPE) though the combination of increased demand and disrupted manufacturing supply chains. As a result of these shortages, many health care providers have temporarily used largely untested, improvised PPE (iPPE). Lack of quality control for makeshift PPE and frequent repurposing of used items to conserve supplies increase both the risk of provider infection and nosocomial spread to uninfected patients. One strategy to reduce risk of infection and preserve existing equipment is the implementation of secondary barrier devices placed directly over patients or providers. The authors describe an inexpensive, disposable, positive-pressure head isolation unit that can be rapidly constructed from materials readily available in nearly all health care settings for under five US dollars. The unit was successfully deployed in Taiwan during the 2003 Severe Acute Respiratory Syndrome (SARS) outbreak, and again during the COVID-19 pandemic. The iPPE worn directly by the health care workers (HCWs) can be donned prior to patient contact in the presence of an air source. This strategy may be more protective than a covering placed over the patient in an aerosol-generating environment, which requires the HCW to be in close contact with the patient prior to securing the protective device.

## Introduction

In May 2003, two resident physicians in a Taiwanese hospital were infected and subsequently died after contracting Severe Acute Respiratory Syndrome (SARS) while intubating a patient who was not known to be infectious. Positive-pressure airway devices, such as powered air-purifying respirators (PAPRs), were cost-prohibitive to implement on a large scale and not widely available during the SARS outbreak. The authors describe an inexpensive, disposable positive-pressure head isolation unit, called a Positive-Pressure Environment Disposable Shield, or PEDS, that was conceived in 2003 to reduce health care worker (HCW) and nosocomial infection risk from exposure to a novel virus.

Aerosol-generating procedures (AGP), such as intubation, are associated with increased risk of virus transmission to HCWs, even when they are wearing appropriate personal protective equipment (PPE).^1,2^ This is particularly concerning for novel and deadly viruses prior to effective therapeutic and vaccine development, since everyone is susceptible and is therefore at risk of infection and subsequent death. While the need for PPE to protect against droplet versus aerosol transmission may initially be unclear for a novel virus,^3^ HCWs performing AGPs must don aerosol protective PPE to ensure adequate protection in either case.

The COVID-19 pandemic has resulted in global shortages of medical supplies including gowns, N-95 respirators, and gloves, even in well-resourced countries. Additionally, health care systems in less-developed countries may have even less access to appropriate PPE for use in decreasing risk of nosocomial viral transmission. Many physicians and independent entities have resorted to constructing improvised PPE (iPPE) and medical devices that can be rapidly produced and used until medical-grade equipment can be replenished.

**Figure 1.**
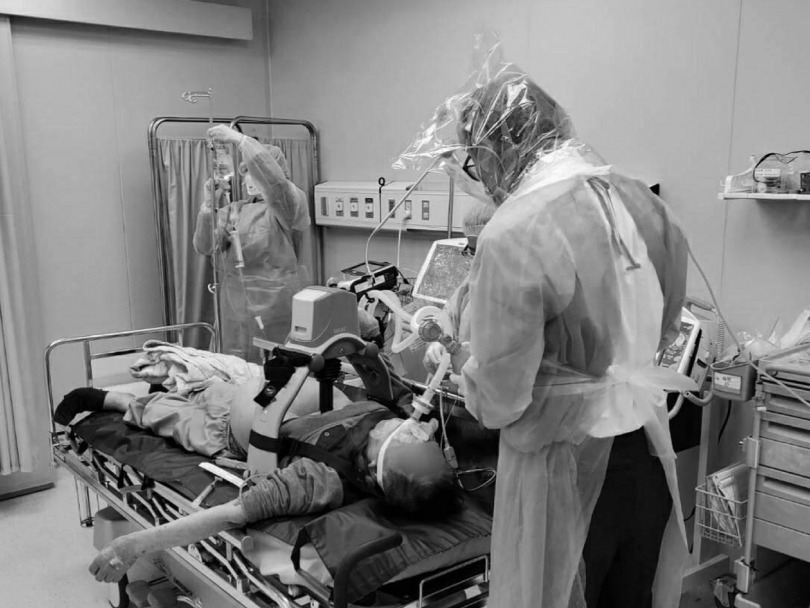
COVID-19 PEDS Head Isolation Unit in Use During an Intubation in Taiwan During the COVID-19 Pandemic.

Note: The intubator is connected to an O_2_ source and is wearing full facial PPE.

Abbreviations: PEDS, Positive-Pressure Environment Disposable Shield; PPE, personal protective equipment.

The PEDS unit, which consists of materials readily available in most prehospital, hospital, and other health care settings, has been successfully tested and implemented in Taiwan during the 2003 SARS outbreaks and again during the COVID-19 pandemic to provide additional protection to HCWs from respiratory viral transmission (Figure 1).

## Report

### Design Overview

**Figure 2.**
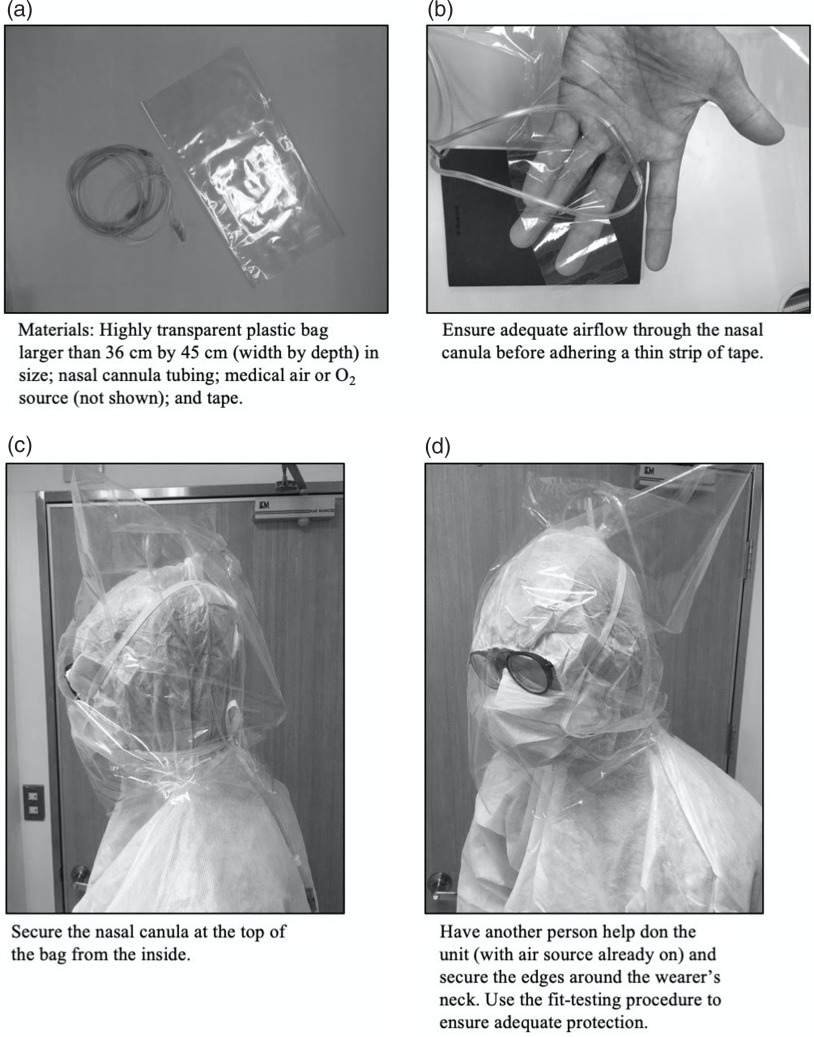
Assembly Instructions for COVID-19 PEDS Head Isolation Unit for HCW Protection.

Abbreviations: HCW, health care worker; PEDS, Positive-Pressure Environment Disposable Shield.

The head unit consists of two main parts: (1) a highly transparent plastic bag larger than 36 cm by 45 cm (width by depth) that is placed over the HCW’s head; and (2) a nasal cannula attached to medical air or oxygen at 10 liters per minute (L/min) (Figure 2A and Figure 2B). Duct tape may be used to create an air-tight seal, further mitigating the risk of exposure on the front and sides of the apparatus; the back should be left open to allow for air expulsion. The nasal cannula tubing is secured from the inside with tape at the top of the apparatus (Figure 2C). Materials needed to construct the device are available in most settings and are very inexpensive with an estimated cost of under five US dollars, at most.

Regulated airflow from the nasal cannula will maintain positive pressure, like a PAPR, while preventing air from being inspired into the head unit during the performance of AGPs. In addition to providing extra protection to the HCW, this leads to less chance for contamination of the underlying PPE. After the procedure is complete, the bag and the nasal cannula can be carefully doffed and discarded into the appropriate medical waste container.

### Fit-Testing Procedure

A modified device fit-testing procedure should be performed after constructing the device to ensure proper protection before entering an aerosolized environment. If available, HCWs should also don an appropriately sized N-95 (or equivalent) respirator, surgical mask, or other facial PPE in addition to this head unit for added protection. Wearing a properly sized N-95 respirator will make the head isolation unit fit testing less necessary since the respirator will already provide sufficient protection for aerosolized droplets should the positive-pressure environment become compromised. In this case, the head isolation unit may be used to primarily reduce risk of N-95 or surgical mask contamination if supplies must be reused due to shortages.

The PAPRs that form a seal around the neck or shoulders usually do not require a fit-testing procedure; however,^4^ the authors recommend a modified procedure to check for leaks and ensure adequate positive air pressure. Select a challenge agent, such as one used for N-95 fit-testing. Ensure the wearer can taste or smell a dilute version of the solution. If the wearer reports no smell or a bitter taste upon direct exposure before fit-testing, use a different solution.^5^ Once a detectable challenge agent has been selected, assemble and generate the positive-pressure environment (Figure 2D). Next, have the tester spray the challenge agent on the wearer from the front, back, and sides of the unit, from less than 30 centimeters away. Wait 30 seconds and ask the wearer if any scent can be detected. If not, then the device has been constructed successfully. If a scent can be detected, wait until the smell or taste dissipates before re-constructing the helmet with a different bag.

### Implementation Considerations

In addition to proper fitment before constructing and implementing the device, HCWs should consider adequacy of air flow to ensure safe and effective use. Compressed medical air, as opposed to medical oxygen (100% O_2_), is preferred for creating the positive-pressure environment, since repeatedly using high-flow oxygen for extended periods of time will greatly increase risk of hyperoxia. Medical oxygen may also not be readily available in large quantities, such as in the prehospital setting. However, medical oxygen is equally effective as compressed air for generating the positive-pressure environment and is acceptable for use on a short-term basis. Sources of compressed air and oxygen include wall outlets or portable cylinders whose gas contents range from 42L in a smaller bottle to 680L in a larger bottle at a pressure of approximately 15,168kPa (2,200 PSI).

To ensure that the apparatus remains sufficiently inflated, even during a sudden inhalation that generates a negative respiratory pressure, the air flow must be sufficiently high at approximately 10L/min. When testing the dimensions listed above, 5L/min was the minimum amount necessary to prevent challenge agent detection; conversely, 15L/min was the upper limit of tolerated flowrate. Other factors that can increase airflow output include widening the air outlet or selecting a larger diameter of tubing. Therefore, for most applications, a rate 10L/min appears to provide sufficient positive pressure while mitigating the risk of discomfort or over-inflation.

Providers who elect to use a mobile air source should be cognizant of the initial available air amount and how much they may need for the length of the procedure. Having inadequate pressure will result in the bag collapsing and compromise of positive-pressure airflow posing a risk of suffocation and PPE contamination. Tubing that is beyond one meter long may also inhibit airflow to the pressurized environment and could present an obstacle for other HCWs in the room. Ideally, HCWs should position themselves near the outlet or the air source. If the provider elects to use a mobile air source, the unit should be properly disinfected before next patient contact.

## Discussion

There is an absence of published data related to the clinical efficacy of COVID-19 iPPE for protection of HCWs and prevention of nosocomial spread. In addition, data are lacking regarding effectiveness of devices created for the primary purpose of PPE preservation. *Ad hoc* implementation of iPPE has created a diverse array of solutions ranging from use of household items to repurposed commercial equipment. Experts have described the existing evidence, implications, and broader context of COVID-related PPE shortages.^6^

While the importance of HCWs wearing PPE is well-understood, protective equipment may also be placed on patients for the same prophylactic purpose.^7^ Notable iPPE devices include the COVID-19 intubation box and inflatable plastic coverings,^8,9^ both of which are patient-centered. One benefit to this approach is that it provides uniform protection for all HCWs in the room. In addition, these devices do not necessarily require fit-testing for the patient since they are designed to isolate aerosolized particles. However, a major disadvantage is that providers may be exposed to aerosols before the iPPE is applied to the patient. Furthermore, patient compliance, supply availability, and large size may present additional challenges.

Conversely, provider-focused PPE represents a familiar option for health care providers in the form of respirators, gowns, and gloves. Provider PPE is a necessity and can only be supplemented by patient-focused PPE – not replaced by it. Popular forms of iPPE include repurposed gloves, mouth and nose coverings, and non-medical respirators to mitigate PPE shortages. Certain materials being used, such as cloth, likely provide inadequate protection against transmittable diseases.^10^ Therefore, it is imperative that any potential improvised provider-focused iPPE not only function well, but also be readily constructed using dependable and preferably disposable materials. The iPPE that utilizes positive pressure may provide additional protection compared to barrier devices that only cover mucosal membranes.^6^

## Limitations

The limitations of the authors’ report are similar to those outlined in other presentations of iPPE. Many constructs are challenging to test due to a variety of factors that include unique assembly techniques, variable materials, difficulty measuring compliance of non-standard implements, and generally increased potential for cross-contamination. In addition, local, state, and national regulations will influence what forms of iPPE can be acceptably implemented in a given situation. All HCWs should ensure they follow guidance from the appropriate authorities before implementing non-standard equipment. Newer clinically verified interventions may also become widespread before PPE supply chains can be replenished, thus mitigating the need for certain innovations.

## Conclusion

The COVID-19 pandemic has necessitated the use of improvised medical equipment to protect both HCWs and patients. Providers in low-resource areas are at especially high risk of contracting and spreading the disease to each other and to patients if PPE is not readily available or must be reused. The simple design and low cost of PEDS, which uses components readily available in nearly all health care settings, means it can be rapidly constructed just prior to patient contact. Also, PEDS has the ability to help preserve valuable stock, such as N-95 respirators, surgical masks, and eye protection in addition to providing a secondary layer of positive-pressure protection for HCWs. Along with standard interventions, such as handwashing and respiratory and contact precautions, iPPE will continue to be a necessary additional line of protection for HCWs in a variety of health care settings globally, until medical supply chains can be replenished. As part of an overall strategy to augment all components of health system surge capacity, the PEDS unit has the potential to help protect and save the lives of frontline HCWs around the globe.

## References

[1] Fowler RA , Guest CB , Lapinsky SE , et al. Transmission of severe acute respiratory syndrome during intubation and mechanical ventilation. Am J Respir Crit Care Med. 2004;169(11):1198–1202. 14990393 10.1164/rccm.200305-715OC

[2] Meng L , Qiu H , Wan L , et al. Intubation and ventilation amid the COVID-19 outbreak: Wuhan’s experience. Anesthesiology. 2020. Epub ahead of print. 10.1097/ALN.0000000000003296 PMC7155908 32195705

[3] Koenig KL . COVID-19: A Call for Science-Informed Management, Evidence Aid. March 3, 2020. https://www.evidenceaid.org/covid-19-a-call-for-science-informed-management/. Accessed April 20, 2020.

[4] Centers for Disease Control and Prevention (CDC). Section 3: Ancillary Respiratory Information. 2020. https://www.cdc.gov/niosh/npptl/topics/respirators/disp_part/respsource3fittest.html. Accessed April 20, 2020.

[5] Occupational Safety and Health Administration (OSHA). 1910.134 App A – Fit Testing Procedures (Mandatory). Published August 4, 2004. https://www.osha.gov/laws-regs/regulations/standardnumber/1910/1910.134AppA. Accessed April 20, 2020.

[6] Livingston L , Desai A , Berkwits M . Sourcing personal protective equipment during the COVID-19 pandemic. JAMA. 2020. Epub ahead of print. 10.1001/jama.2020.5317 32221579

[7] US Food and Drug Administration. Personal Protective Equipment for Infection Control. Published February 10, 2020. https://www.fda.gov/medical-devices/general-hospital-devices-and-supplies/personal-protective-equipment-infection-control. Accessed April 30, 2020.

[8] Aerosol Block. Design Roadmap. https://www.aerosolblock.org/feature-designs/design-roadmap. Accessed April 29, 2020.

[9] YouTube. Larry Mellick, MD. Safer HFNC and the COVID-19 Patient, Part II. https://www.youtube.com/watch?v=yyZbF3_tlAA&t=56s. Accessed April 29, 2020.

[10] MacIntyre CR , Seale H , Dung TC , et al. A cluster of randomized trial of cloth masks compared with medical masks in health care workers. BMJ Open. 2015;5(4):e006577. 10.1136/bmjopen-2014-006577 PMC4420971 25903751

